# Decision making and implicit suicidality in schizophrenia spectrum disorders

**DOI:** 10.1192/j.eurpsy.2021.2134

**Published:** 2021-08-13

**Authors:** O. Vorontsova, T. Medvedeva, S. Enikolopov, O. Boyko, G. Rupchev

**Affiliations:** 1 Clinical Psychology, Federal State Budgetary Scientific institution “Mental health research center”, Moscow, Russian Federation; 2 Laboratory Of Psychopharmacology, FSBSI Mental Health Research Center, Moscow, Russian Federation

**Keywords:** Rorschach test, Iowa gambling task, schizophrénia, implicit suicidality

## Abstract

**Introduction:**

Research shows that mental illnesses increase suicidal risk. Studies have found that suicidal risk is associated with impaired decision-making.

**Objectives:**

To analyze decision making based on emotional learning in implicit suicidality.

**Methods:**

56 male patients with schizophrenia spectrum disorder (F20) and denied presence of suicidal ideation were involved into the study (mean age 23±2.7),. Methods: Iowa gambling task (IGT) - integral indicators were used: the prevalence of “good” choices over “bad” ones, total score; and an indicator reflecting the ignorance of consequences of one’s choice - the subject remains on a “bad deck” after a loss. The Rorschach test (Rorschach Comprehensive system) was administered: Suicide Constellation «S-CON» and its components were used.

**Results:**

According to the analysis, prevalence of “good” choices (IGT) negatively correlates with «S-CON» (Spearman’s correlation -0.328*, hereinafter significance level: ~ - p<0.1; * - p<0.05). A decrease in the total IGT score is associated with the following cognitive indicators: disregard for social conventions, non-conformism («P») (Spearman’s, 0.337*); a tendency to react defensively to a problem situation, blocking activity in terms of making decisions («R») (0.308*). Ignoring the consequences of one’s choice (IGT) correlates with such emotional factors as emotional incontinence, superficiality of emotions, emotional lability («FC:CF+C») (-.0382*), ambivalence of emotions («Blcol-shd») (statistical tendency, 0.277~), expressed dissatisfaction with the existing situation, internal tension and dysphoria («S») (0.291~).

**Conclusions:**

The relationship of implicit suicidality with decision-making was found to be similar to the relationship of pronounced suicidality with decision-making. Suicidality is associated with impaired ability to make decisions based on emotional learning.

**Disclosure:**

No significant relationships.

